# Mindfulness Practice and Work Performance: The Mediating Role of Emotional Intelligence and Psychological Capital

**DOI:** 10.1002/brb3.70291

**Published:** 2025-01-28

**Authors:** Hoang Cuu Long, Ho Minh An, Pham Thi Phuong Dung, Nguyen Le Dinh Quy

**Affiliations:** ^1^ School of International Business‐Marketing University of Economics Ho Chi Minh City ‐ UEH Ho Chi Minh City Vietnam; ^2^ Hung Vuong University of Ho Chi Minh City Ho Chi Minh City Vietnam; ^3^ Journalism and Communications School University of Social Sciences and Humanities, Vietnam National University Hanoi Vietnam

**Keywords:** emotional intelligence, job performance, mindfulness‐to‐meaning (MTM), mindfulness, psychological capital

## Abstract

**Purpose:**

The practice of mindfulness is becoming more widespread among employees, with potential benefits for workplace outcomes. However, there is a paucity of research on the mechanisms linking mindfulness to job performance.

**Method:**

This study investigated the mediating functions of emotional intelligence and psychological capital in the relationship between mindfulness and job performance among 263 office employees in Ho Chi Minh City, Vietnam. The “mindfulness‐to‐meaning” viewpoint posits that through the practice of mindfulness, people are able to transcend negative emotions, gain a deeper understanding of themselves, and progress more intentionally by living more consciously in the present moment. Participants completed questionnaires measuring mindfulness, emotional intelligence, psychological capital, and occupational performance.

**Finding:**

According to the results of structural equation modeling, mindfulness has a direct positive influence on job performance. In addition, emotional intelligence partially mediated the relationship between mindfulness and performance, as evidenced by a greater indirect effect than direct effect. However, the indirect effect via psychological capital was less significant than the direct effect of mindfulness. These findings identify emotional intelligence as a key personal resource that transmits the positive effects of mindfulness on workplace performance.

**Conclusion:**

The results advance comprehension of intermediary pathways in mindfulness research grounded in the resource‐based view. Integrating emotional intelligence training into mindfulness programs could increase employee productivity in organizations.

## Graphical abstract

Emotional intelligence as a key personal resource that transmits the positive effects of mindfulness on workplace performance. The results advance comprehension of intermediary pathways in mindfulness research grounded in the resource‐based view. Integrating emotional intelligence training into mindfulness programs could increase employee productivity in organizations.

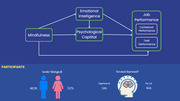

## 1 Introduction

Mindfulness (MF) has become increasingly popular, with evidence showing it enhances personal health (Wang and Lei 2023; Dai et al. 2022) and workplace outcomes (Shahbaz and Parker 2022; Johnson, Park, and Chaudhuri). However, the mechanisms linking MF to work performance remain underexplored (Dane 2011; Good et al. 2016). This research aims to investigate the role of emotional intelligence (EI) and psychological capital (PsyCap) in mediating the relationship between MF and work performance.

*MF* involves focusing on the present moment in a non‐judgmental way (Siegel, Germer, and Olendzki n.d.). It has been associated with improved mental health (Berdida, Lopez, and Grande 2023; Ibrahim et al. 2022), stress reduction (Wang et al. 2023; Mesquita et al. 2023), and emotional regulation (Ng et al. 2023). MF can reduce anxiety, depression, and rumination (Pressman et al. 2017; Syeda and Andrews 2021; Enkema et al. 2020) and improve work performance by promoting calm responses to stress and enhancing focus and resilience.

*EI* refers to the ability to perceive, understand, and manage emotions (Mayer and Salovey 1997). EI has been shown to positively impact work performance by improving interpersonal relationships and reducing stress (Deshpande and Srivastava 2023; Shakeel and Khan 2023; Alsufyani et al. 2022). High EI leads to better conflict resolution, communication, and adaptability, which improves work performance (Chauhan, Kaul, and Maheshwari 2022; Jeong and Lee 2022). Moreover, EI has been linked to improved mental health, reducing stress and anxiety (Jacobs and Wollny 2022; Moeller, Seehuus, and Peisch 2020).

*PsyCap* includes hope, self‐confidence, resilience, and optimism (Luthans, Youssef, and Avolio 2007). It enhances motivation, job satisfaction, and work performance (Jiao et al. 2022; Bramantya and Muafi 2022) and promotes resilience in challenging situations (Shan, Ishak, and Fan 2022). High PsyCap leads to improved work outcomes, job satisfaction, and mental health (Sood and Puri 2022; Finch, Farrell, and Waters 2020).

MF can improve emotional regulation (Himes et al. 2021), which enhances EI and boosts PsyCap (Gordani and Sadeghzadeh 2023). Therefore, EI and PsyCap are essential mechanisms linking MF to work performance. Despite theoretical support, empirical studies on their role as mediators are limited. Understanding these mechanisms will inform workplace interventions to improve performance and mental health, especially in cities like Ho Chi Minh, Vietnam.

### 1.1 MF and Work Performance

MF can positively impact work performance by improving stress management, enhancing job satisfaction, and increasing organizational commitment (Lin et al. 2022; Song, Pan, and Wang 2021). Studies show that MF increases performance in various sectors, including healthcare (Janssen et al. 2020) and frontline services during the COVID‐19 pandemic (Xie, Ifie, and Gruber 2022). MF reduces stress, boosts problem‐solving ability, and strengthens emotional management, leading to higher work performance (Tiwari and Garg 2019).

Hypothesis 1 **(H1)**. *Mindfulness will have a positive effect on job performance*.

### 1.2 MF and EI

MF has been shown to enhance EI by improving emotional awareness and regulation (Kou et al. 2022). Research indicates that MF increases EI, which helps individuals manage emotions and cope with stress in the workplace (Xie et al. 2021; Xiang et al. 2021). By improving EI, MF can enhance interpersonal relationships and emotional regulation, leading to better work performance (Schutte, Keng, and Cheung 2021).

Hypothesis 2 **(H2)**. *Mindfulness will have a positive effect on emotional intelligence*.

### 1.3 EI and Job Performance (JP)

EI is crucial for work performance, with high EI improving conflict resolution, communication, and teamwork (Sharma and Tiwari 2023; Mittal 2021). Studies show that EI positively correlates with work performance in diverse fields such as healthcare (Huang et al. 2022) and hospitality (Alonazi 2020). EI helps employees manage stress and navigate challenges, leading to improved job satisfaction and performance (Chauhan, Kaul, and Maheshwari 2022).

Hypothesis 3 **(H3)**. *Emotional intelligence will have a positive effect on job performance*.

Hypothesis 4 **(H4)**. *Emotional intelligence mediates the relationship between mindfulness and job performance*.

### 1.4 MF and PsyCap

MF practices enhance PsyCap by increasing resilience, optimism, and self‐confidence (Kotzé 2018; Fiaz and Muhammad Fahim 2023). MF has been shown to increase work engagement and satisfaction, fostering PsyCap and improving overall career success (Zhu et al. 2021). As a mediator, MF can enhance PsyCap, leading to improved work performance and well‐being (Ali et al. 2022).

Hypothesis 5 **(H5)**. *Mindfulness will have a positive effect on psychological capital*.

### 1.5 EI and PsyCap

EI contributes to the development of PsyCap by improving emotional regulation and promoting positive psychological states such as hope and optimism (Lye et al. 2023; Fei et al. 2023). Studies suggest that EI can enhance PsyCap, reducing anxiety and improving work performance and well‐being (Liao, Hu, and Huang 2022). EI and PsyCap work together to boost work performance and resilience in challenging situations (Mjoli and Aderibigbe 2019; Gong, Chen, and Wang 2019).

Hypothesis 6 **(H6)**. *Emotional intelligence will have a positive effect on psychological capital*.

### 1.6 PsyCap and JP

PsyCap has been shown to improve work performance by fostering motivation, resilience, and optimism (Sarwar et al. 2023; Paliga et al. 2022). Employees with high PsyCap are more engaged, adaptable, and productive, contributing to better JP across various industries (Huang et al. 2021). PsyCap also enhances employee well‐being, reducing stress and improving job satisfaction (Tüzün, Çetin, and Basim 2018).

Hypothesis 7 **(H7)**. *Psychological capital will have a positive effect on job performance*.

Hypothesis 8 **(H8)**. *Psychological capital mediates the relationship between mindfulness and job performance*.

**FIGURE 1:**
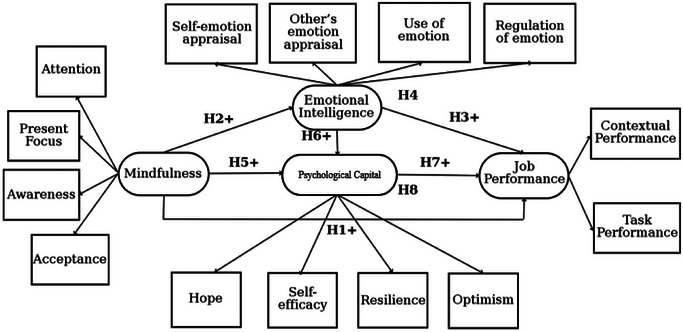
Proposed conceptual model.

The proposed conceptual model is illustrated in Figure 1, providing an overview of the factors and their relationships.

## 2 Materials and Methods

### 2.1 Methodology

#### 2.1.1 Study Design

This study employed a cross‐sectional survey approach to investigate the correlation between MF practices and work performance, emphasizing the mediating effects of EI and PsyCap. The research focused on full‐time office employees in Ho Chi Minh City, Vietnam, as this demographic is particularly pertinent for examining the influence of MF on JP outcomes in a professional context.

#### 2.1.2 Sampling Method and Population Justification

Convenience sampling was utilized to recruit participants from various municipal offices in Ho Chi Minh City. Office employees were chosen as the study concentrated on the impact of MF and emotional variables on performance in organized work settings. A sample size of 263 participants was established on the basis of previous research with analogous designs, ensuring sufficient statistical power to identify significant connections among study variables.

#### 2.1.3 Inclusion and Exclusion Criteria

Eligibility for participation was granted to full‐time office employees aged 20 or older who freely volunteered to partake in the study. Individuals engaged in temporary or non‐office positions were excluded, as their work environments may substantially differ from those of typical office employees, thereby influencing the study variables.

#### 2.1.4 Data Collection and Sociodemographic Variables

The data collection utilized a self‐administered questionnaire disseminated to employees at the participating offices. The forward‐backward translation method was employed to guarantee cultural and linguistic precision for Vietnamese participants. Demographic data, encompassing age, gender, marital status, education level, and work experience, were collected to furnish contextual background and facilitate subgroup analysis within the data.

### 2.2 Measurement

Data were gathered through a structured, *self‐administered* questionnaire to safeguard participant confidentiality and reduce interviewer bias. The subsequent scales were utilized.

#### 2.2.1 “MF Practice” Variable

MF is assessed using the Cognitive and Affective Mindfulness Scale—Revised (CAMS‐R) developed by Feldman et al. (2007), which consists of 12 observed characteristics categorized into 4 groups: *attention* (Attn), *present focus* (PreFo), *awareness* (Awa), and *acceptance* (Acc). The scale consists of 12 items covering attention, present focus, awareness, and acceptance. Participants rated each item on a 5‐point Likert scale (1 = strongly disagree to 5 = strongly agree). Moreover, this scale has been validated in previous studies, demonstrating high reliability with Cronbach's alpha values exceeding 0.70 (Huang et al. 2022; Lima et al. 2020; Whitaker et al. 2019) (see Table A1).

#### 2.2.2 “EI” Variable

EI was assessed with the Wong and Law Emotional Intelligence Scale (WLEIS), comprising 16 items distributed across 4 dimensions: self‐emotion appraisal, others’ emotion appraisal, use of emotion, and regulation of emotion. Responses were additionally documented using a 5‐point Likert scale. This scale has demonstrated robust reliability and validity in previous studies, with Cronbach's alpha generally exceeding 0.80 (Pan, Wu, and Zhang 2022; George, Okon, and Akaighe 2023; Gong, Chen, and Wang 2019; Iliceto and Fino 2017) (see Table A2).

#### 2.2.3 “PsyCap” Variable

The Psychological Capital Questionnaire (PCQ‐24) was utilized to evaluate PsyCap, consisting of 24 items that reflect hope, self‐efficacy, resilience, and optimism. Every item was evaluated using a 5‐point Likert scale. The PCQ‐24 exhibits strong internal consistency across multiple trials, frequently achieving Cronbach's alpha values more than 0.85 (Dirzyte, Perminas, and Biliuniene 2021; Gong, Chen, and Wang 2019; Tang, Shao, and Chen 2019; Li 2018) (see Table A3).

#### 2.2.4 Job Performance

JP was assessed via a modified scale from Borman and Motowidlo (1993), encompassing both task performance and contextual performance. Responses were documented using a 5‐point Likert scale, which has demonstrated accurate psychometric features in prior research, with Cronbach's alpha values exceeding 0.80 (Thuy and Phinaitrup 2023; Hameed, Khwaja, and Zaman 2023; Aboagye, Dai, and Bakpa 2022; Bhatti, Mat, and Juhari 2018).

The validity and reliability of each scale were established in prior investigations, confirming their appropriateness for this research situation. The incorporation of these dependable instruments establishes a solid basis for assessing the influence of MF on JP, mediated via EI and PsyCap (see Table A4).

#### 2.2.5 Data Analysis

**FIGURE 2:**
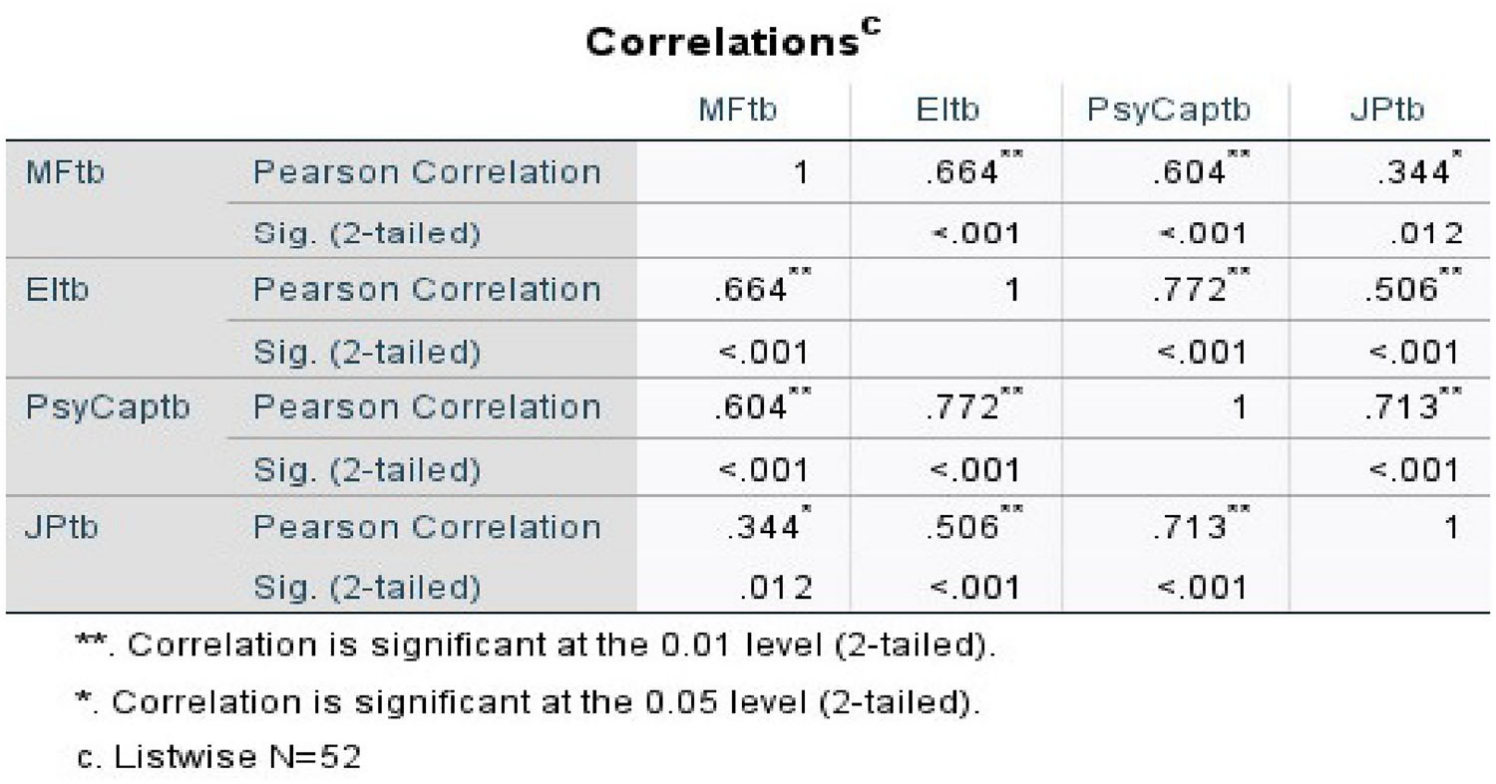
Correlation coefficient among variables.

The data acquired for this study was analyzed using SPSS version 27 and Smart PLS version 4.0.9.5. During the pilot test phase, the author utilized SPSS to assess and verify the quality of the 52 samples. The analysis revealed a strong likelihood of significant multicollinearity due to the correlation coefficient. The model has a strong correlation among variables, as seen in Figure 2. The majority of correlations are over 0.5, with some reaching as high as 0.7 (Hair et al. 2022). The author attributes this example to three factors: (1) respondents providing identical replies, (2) inaccurate design of the scale questions with repetitive content, and (3) an excessive number of observed variables in the study topics.

In order to address these issues, the author will initially categorize the observed variables of each study idea into smaller groups based on their hypothesis, specifically to address problem (3). To address the issue of multicollinearity, the author employs advanced constructions that are more complex in nature (Hair and Sarstedt 2021). Furthermore, the author employed the forward–backward approach to translate the scale, specifically to address Difficulties 2 and 3. This method involves translating the scale both forward and backward to assure the preservation of the question's accurate meaning.

Once the majority of mistakes have been reduced, the sample data will undergo descriptive statistical analysis. Subsequently, it will be inputted into Smart PLS software for analysis using the partial least squares structural model (PLS‐SEM) with the 10,000‐bootstrap procedure. This analysis will be conducted in two parts: (1) Assess the measurement model to determine the reliability of observed variables, internal consistency reliability, convergent validity, and discrimination of research concepts in the model; (2) evaluate the structural model by conducting multicollinearity testing, assessing hypotheses based on the level of significance and appropriateness of the model, and evaluating its explanatory ability and the model's ability to accurately predict outcomes that are not included in the original dataset.

According to Hair et al. (2022), the researchers choose PLS‐SEM as the method to analyze and evaluate the hypotheses in this study due to the following justifications:

- Appropriate for models that involve several research concepts and intricate structures (such as quadratic variable models).
- If the variables deviate from the principle of normal distribution, PLS‐SEM is minimally impacted but may still generate precise analysis outcomes.
- It is feasible to forecast the optimization of explained variance in the dependent variables of the research model.

### 2.3 Ethical Consideration

This research was executed in alignment with ethical standards to guarantee participant autonomy and data confidentiality. Before data collection, the study protocol received approval from the ethics committee at the University of Economics Ho Chi Minh City (UEH), confirming compliance with ethical norms. All participants granted informed consent and were apprised of their ability to withdraw from the study at any moment. Furthermore, all data were anonymized to safeguard participant names, and responses were securely archived to ensure confidentiality. In conducting this investigation, we adhered to the STROBE (Strengthening the Reporting of Observational Studies in Epidemiology) standards, which advocate for thorough and honest reporting in observational research. Compliance with these principles ensures the correct and clear presentation of the study's methodology, conclusions, and consequences.

## 3 Results

### 3.1 Descriptive Statistics

As demonstrated in Table 1, 135 of the 263 research samples were women, or over 51.7%. Men made up 48.3% of the remaining 127 samples. There are five major age groups in the study, each lasting 5 years. No significant age disparities exist in the distribution of individuals among these categories. The age group of 20‐ to 25‐year‐olds who have recently graduated and are employed has the lowest representation, with only 53 people (20.2%). The largest age group is above 36, with 85 participants (32.3%). There are 65 (24.7%) and 60 (22.8%) people in the remaining age groups 26–30 and 31–35. Similar to “gender,” “unmarried” accounts for 51% or 134 people. The remaining 129 are “married” (49%). Bachelor's degree holders make up 46.8% of the population, with 123 people. Recent high school graduates have the fewest qualifications, with only 10 representatives (3.8%). The 263 survey participants included 33 college/intermediate students (12.5%). The survey's largest participation rate comes from “postgraduate” degree holders (97 respondents). This category makes up 36.9% of responders, second only to bachelor's degree holders.

**TABLE 1:** Descriptive statistics (*N* = 263).

Variable	Observable variable	*N*	%
Sex (biological)	Male	127	48.3
	Female	136	51.7
Aging	20–25	53	20.2
	26–30	65	24.7
	31–35	60	22.8
	> 36	85	32.3
Marriage status	Single	134	51
	Married	129	49
Education	High school	10	3.8
	Intermediate	33	12.5
	Bachelor	123	46.8
	Master/PhD	97	36.9
Working experience	≤ 5 years	78	29.7
	6–10 years	76	28.9
	11–15 years	25	9.5
	Above 15 years	84	31.9
Stressed/depressed?	Not yet	121	46
	Experienced	142	54

The variable “years of experience” is split into 5‐year intervals like “education level.” About 31.9% of the population—84 people—have more than 15 years of professional experience. Next are those with “less than 5 years of work experience” (78, 29.7%) and “6–10 years of work experience” (76, 28.9%). Only 9.5% of 25 had “11–15 years of work experience.”

Overall, 54% of the 263 poll respondents stated their jobs had caused stress or depression. Overall, 46% said they have never experienced this.

### 3.2 Measurement Model

#### 3.2.1 Indicator Reliability

On the basis of the data shown in Table A5, the author has determined that the research idea “Cognitive and Emotional MF” has the lowest values for Cronbach's alpha (0.916) and composite reliability (0.932). On the other hand, the research concept “PsyCap” has the highest values for Cronbach's alpha (0.969) and composite reliability (0.972). Furthermore, the reliability coefficient, referred to as roh_a, typically falls between the Cronbach's alpha coefficient and composite reliability. It serves as a satisfactory compromise between these two approaches. A study conducted by Hair et al. (2019) revealed high internal consistency reliability, with roh_a values ranging from 0.917 to 0.970.

According to this score, the author can infer that all research notions in the model meet the criteria for internal consistency reliability. However, when the Cronbach's alpha coefficient and composite reliability exceed 0.9, an issue arises indicating the presence of semantically redundant items that express the same phrase again. Referenced (Drolet and Morrison 2001; Hayduk and Littvay 2012). In order to address this issue, the author must assess the convergent and discriminant validity of the scales in the subsequent stages.

#### 3.2.2 Convergent Validity

- “MF” concept

The average variance extracted (AVE) index of the latent variables Attn, Awa, and Acc exceeds 0.5, particularly measuring at 0.782, 0.799, and 0.757, respectively. Regarding the latent variable PreFo, it does not have an AVE index as just one observed variable remains. Two‐thirds of the observed variables were deleted during the factor loading process. The AVE index for the MF research idea is 0.631, which is above the threshold of 0.50, indicating that it successfully meets the criteria for convergent validity.

- “EI” concept

The AVE index for the latent variables SEA, OEA, UOE, and ROE exceeds 0.5, particularly measuring 0.752, 0.734, 0.779, and 0.691, respectively. The AVE index for the EI research idea is 0.614, which is above the threshold of 0.50, indicating that it successfully meets the criteria for convergent validity.

- “PsyCap” concept

The AVE index for the latent variables HOPE, SELF, RESI, and OPT is all above 0.5, particularly 0.680, 0.668, 0.664, and 0.780. The AVE index for the PsyCap is 0.620, which is above the threshold of 0.50, indicating that it successfully meets the criteria for convergent validity.

- “JP” concept

The AVE index of the latent variables CP and TP exceeds 0.5, particularly measuring 0.654 and 0.649, respectively. The AVE index for the JP is 0.621, which is above the threshold of 0.50, indicating that it successfully meets the criteria for convergent validity.

#### 3.2.3 Discriminant Validity

On the basis of the findings in Table 2, the author determined that the HTMT index of all concept pairings is less than 0.90. This indicates that the research concepts have successfully passed the discriminant validity test, meaning that clear distinctions have been formed for the research concepts in the proposed research model.

**TABLE 2:** Testing discriminant validity through HTMT index.

	EI	JP	MF
JP	0.832		
MF	0.881	0.831	
PsyCap	0.780	0.825	0.783

Abbreviations: EI, emotional intelligence; JP, job performance; MF, mindfulness; PsyCap, psychological capital.

### 3.3 Structural Model

#### 3.3.1 Multicollinearity Testing

During the initial stage of assessing the structural model, the author employs the variance inflation factor (VIF) to ascertain the level of correlation among variables in the model, which is also referred to as testing. In order to prevent the occurrence of multicollinearity problems and their impact on the structural model, it is necessary for the VIF index to be below 5 and ideally below 3 (Hair et al. 2022).

The VIF coefficients in Table 3 are all below 5, falling within the range of 1–3.652. Thus, multicollinearity is absent.

**TABLE 3:** Assessing multicollinearity through variance inflation factor.

	EI	JP	PsyCap
EI		3.652	3.135
MF	1	3.478	3.135
PsyCap		2.553	

Abbreviations: EI, emotional intelligence; JP, job performance; MF, mindfulness; PsyCap, psychological capital.

#### 3.3.2 Testing Hypotheses

For a two‐tailed test, where the statistical significance threshold is set at 0.05 or 5%, the critical value is determined to be 1.96 (Hair et al. 2022). Upon examining Table 4, the author observes that the *t*‐test indices are above the critical threshold, indicating that all path coefficients of the latent variables in the research model are statistically significant at a 95% confidence level. The assumptions on the link among research concepts suggested are accepted.

**TABLE 4:** Path coefficient values with bootstrapping technique (*N* = 10,000).

	Original sample (O)	Sample mean (M)	Standard deviation	*T* statistics (|O/STDEV|)	*p* values
H1: MF → JP	0.238	0.238	0.079	3.002	0.003
H2: MF → EI	0.825	0.826	0.025	32.838	0.000
H3: EI → JP	0.311	0.305	0.071	4.371	0.000
H5: MF → PsyCap	0.366	0.369	0.086	4.244	0.000
H6: EI → PsyCap	0.450	0.447	0.083	5.400	0.000
H7: PsyCap → JP	0.384	0.389	0.091	4.224	0.000

Abbreviations: EI, emotional intelligence; JP, job performance; MF, mindfulness; PsyCap, psychological capital.

The analysis findings indicate that there is a statistically significant influence of MF on JP (*β* = 0.238, *p* = 0.003), on EI (*β* = 0.825, *p* = 0.000), and on PsyCap (*β* = 0.366, *p* = 0.000). Statistical analysis was conducted, confirming H1, H2, and H5. In addition, the influence of EI on JP (*β* = 0.311, *p* = 0.000) and PsyCap (*β* = 0.450, *p* = 0.000) is also statistically significant. On the basis of statistical analysis, H3 and H6 are accepted. The statistical analysis confirms that there is a substantial influence of PsyCap on JP, as indicated by the coefficient *β* = 0.384 and *p* value of 0.000. Therefore, H7 is accepted.

In order to verify the conclusion mentioned above, the author used the bootstrap confidence interval to examine if the path coefficient differs substantially from 0. Confidence intervals offer insights into the reliability of the calculated coefficient by presenting a range of likely population values for the parameter, which is influenced by the variability of the data and the sample size (Hair et al. 2022). The bootstrap confidence interval is calculated using the standard deviations obtained from the bootstrapping procedure. It represents the range within which the real population parameter is likely to fall, given a specified degree of confidence (e.g., 95%). Thus, if the confidence interval for the path coefficient does not encompass zero, the null hypothesis that the path is zero will be refuted, indicating that the path coefficient has a statistically significant impact.

Upon Table 5, the author can reaffirm that all of the aforementioned path coefficients are statistically significant at the 95% level. This is because the confidence intervals of these path coefficients do not encompass the value 0.

**TABLE 5:** Confidence interval bootstrapping.

Hypothesis	Original sample (O)	Sample mean (M)	2.5%	97.5%	Results
H1: MF → JP	0.238	0.238	0.084	0.393	Accept
H2: MF → EI	0.825	0.826	0.772	0.870	Accept
H3: EI → JP	0.311	0.305	0.164	0.443	Accept
H5: MF → PsyCap	0.366	0.369	0.195	0.534	Accept
H6: EI → PsyCap	0.450	0.447	0.284	0.608	Accept
H7: PsyCap → JP	0.384	0.389	0.220	0.573	Accept

Abbreviations: EI, emotional intelligence; JP, job performance; MF, mindfulness; PsyCap, psychological capital.

### 3.4 Mediating Role Assessment

In order to examine the mediating impact of the variables EI and PsyCap, a two‐stage process proposed by Preacher and Hayes (2004, 2008, 2004, 2008) will be followed. The first phase involves assessing the indirect influence of MF on JP through the mediating variables EI and PsyCap. Once PsyCap has been enhanced, the next phase involves reassessing the direct influence of MF on JP by examining the path coefficient.

On the basis of the findings in Table 6, it is evident that the indirect effects from MF to JP through EI (*β* = 0.257) and PsyCap (*β* = 0.140) are statistically significant at the 95% confidence range. This is because these indirect effects do not contain the value 0. Moreover, the findings indicate that MF has a statistically significant and beneficial effect on JP, as evidenced by a standardized regression coefficient of *β* = 0.238. Hence, it can be inferred that EI and PsyCap partially mediate the connection between MF and JP. This conclusion is supported by the statistical significance of both the indirect and direct effects. Additionally, the product of these effects (0.257 × 0.238 = 0.061; 0.140 × 0.238 = 0.033) demonstrates that they are aligned in the same direction, as reported by Hair and Sarstedt (2021). To clarify, we accept two hypotheses, namely, H4 and H8.

**TABLE 6:** Assessing direct and indirect impacts from mindfulness (MF) to job performance (JP) through mediating variables emotional intelligence (EI) and psychological capital (PsyCap).

Path coefficients	Direct impact	95% interval confidence of direct impact	Significant (*p* < 0.05)	Indirect impact	95% interval confidence of indirect impact	Significant (*p* < 0.05)
MF → JP	0.238	[0.084; 0.393]	Yes	0.257 *(throughout EI)*	[0.137; 0.364]	Yes
				0.140 *(throughout PsyCap)*	[0.060; 0.255]	Yes
				0.142 *(throughout both EI and PsyCap)*	[0.068; 0.243]	Yes

### 3.5 Model's Explanatory Power

On the basis of the information provided in Table 7, it can be concluded that MF is responsible for explaining 68% and 60.5% of the variation (variance) in EI and PsyCap, respectively. The remaining percentage represents mistakes. Derived from exogenous variables and external influences. Similarly, the variable JP is influenced by multiple independent variables, which can be represented by arrows. It can be stated that JP accounts for 73.4% of the variation or variance explained by the independent variables (MF, EI, and PsyCap). The remaining variation is attributed to systematic errors and other factors not included in the model.

**TABLE 7:** The coefficient of determination (*R* ^2^).

Research concept	*R* ^2^	*R* ^2^ adjusted
EI	0.681	0.680
PsyCap	0.608	0.605
JP	0.737	0.734

Abbreviations: EI, emotional intelligence; JP, job performance; PsyCap, psychological capital.

If *R* ^2^ is employed to assess the influence of an exogenous variable on an endogenous variable, the effect size coefficient *f* ^2^ is utilized to evaluate the alteration in *R* ^2^ when the exogenous variable is excluded from the research model. The citation is from the publication by Hair et al. (2022). Cohen (1998) states that the impact of external factors will be significant if *f* ^2^ > 0.35, moderate if *f* ^2^ ≥ 0.15, and minimal if *f* ^2^ ≥ 0.02 (numbers below 0.02 indicate no influence whatsoever).

According to the data in Table 8 , the author concludes that the exogenous variable MF has a significant impact on EI (2.135) but only a little impact on PsyCap (0.109) and JP (0.062). The EI variable exerts a considerable impact on PsyCap (0.165), whereas its impact on JP is minimal (0.101). Ultimately, the impact of PsyCap on JP is somewhat significant, with a coefficient of 0.219.

**TABLE 8:** Effect size *f* ^2^.

	EI	JP	PsyCap
EI		0.101	0.165
MF	2.135	0.062	0.109
PsyCap		0.219	

Abbreviations: EI, emotional intelligence; JP, job performance; MF, mindfulness; PsyCap, psychological capital.

## 4 Discussion

This study provides substantial insights into the correlation between MF practices and JP, indicating that EI and PsyCap operate as partial mediators in this relationship. The findings corroborate the mindfulness‐to‐meaning theory (MMT), which posits that MF practices facilitate good psychological outcomes by improving emotional and cognitive processing (Garland et al. 2015). MF enhances EI and PsyCap, hence cultivating adaptive capacities that improve work performance (Xie, Ifie, and Gruber 2022; Kotzé 2018).

The study's results demonstrate that EI serves as a more potent mediator than PsyCap in the correlation between MF and job success. This aligns with research indicating that MF training improves emotional awareness and regulation, fundamental aspects of EI, which positively affect work outcomes by fostering empathy, interpersonal skills, and self‐regulation (Schutte, Keng, and Cheung 2021; Xiang et al. 2021). Elevated EI enables people to navigate workplace pressures and sustain constructive relationships, hence immediately enhancing JP (Oh and Jang 2020; Deshpande and Srivastava 2023).

Conversely, although PsyCap is substantially linked to MF and work performance, it functions as a less robust mediator. This aligns with the research of Bi and Ye (2021) and Fiaz and Muhammad Fahim (2023), indicating that PsyCap enhances resilience, optimism, and self‐efficacy, hence assisting employees in surmounting professional problems. Nonetheless, the indirect impact of PsyCap is comparatively diminished, perhaps due to the direct advantages of EI—such as emotional control and social awareness—that are more directly pertinent to performance results (Lye et al. 2023; Bockorny and Youssef‐Morgan 2019).

These findings highlight the practical significance of MF therapies in improving employee performance. Organizations may gain advantages by integrating EI training into MF programs, focusing on competencies such as empathy and self‐regulation. Studies indicate that workplace initiatives centered on EI enhance employees’ stress resilience and problem‐solving abilities, correlating with heightened job satisfaction and performance (Coleman and Coleman 2019; Sharma and Tiwari 2023). By cultivating an emotionally friendly workplace, such initiatives can yield enduring enhancements in productivity and employee welfare.

This study indicates that although PsyCap possesses beneficial attributes (optimism, resilience), its effect on performance may necessitate enhanced organizational support or leadership involvement to achieve maximum efficacy (Sarwar et al. 2023; Paliga et al. 2022). This insight indicates that organizations could enhance PsyCap by fostering a supportive culture that cultivates employees’ hope, resilience, and confidence.

These discoveries substantially enhance the existing literature on MF in professional environments by distinguishing the mediation functions of EI and PsyCap. This study elucidates the specific mechanisms by which MF affects JP, offering a refined comprehension of the impact of MF‐based therapies on workplace outcomes (Janssen et al. 2020; Xie et al. 2021). Future study may investigate supplementary mediators, such as stress management competencies or workplace connections, to further clarify the impact of MF on JP across various occupational contexts.

### 4.1 Limitations

Similar to other studies, this study has limitations that offer opportunities for further research. Initially, this study was conducted using cross‐sectional data, which restricts the ability to draw definitive conclusions about the cause‐and‐effect linkages and analyze patterns over time. Because there is insufficient information on the chronological order of variables (Levin 2006). Future research can include longitudinal or experimental investigations to ascertain causal linkages and temporal sequencing.

Furthermore, the use of self‐report studies may give rise to problems regarding response bias, namely, social‐desirability bias. Hence, future research endeavors might integrate additional impartial evaluations of JP and MF.

Additionally, the study's sample set specifically consisted of Vietnamese office workers, which may restrict the applicability of the findings to different cultures and professions. Hence, it is advisable for future study to examine the suitability of the aforementioned findings in other situations and businesses. Ultimately, this study solely focused on investigating EI and PsyCap as mediators. Future study may explore additional factors, such as stress, burnout, and work–life balance, which are relevant to the topic.

Further investigation aimed at addressing these constraints will enhance the comprehension of the extent to which MF enhances performance and the mechanisms via which it does so. Nevertheless, the present study represents a significant advancement in understanding the processes by which MF improves JP and well‐being.

## 5 Conclusion

### 5.1 Theoretical Implication

This study extends the literature by furthering the MMT, which asserts that MF augments psychological resources, resulting in improved life and job outcomes. The study demonstrates that EI and PsyCap partially mediate the association between MF and JP, highlighting the unique functions of these mediators in converting MF into professional advantages. EI has surfaced as a key mediator, indicating that MF may be particularly beneficial in improving self‐regulatory and interpersonal skills, which are essential for professional success. PsyCap, however significant, showed a reduced direct influence, suggesting that optimism and resilience, while crucial, may be most effective when augmented by EI in high‐performance settings.

The findings of this study necessitate further research to investigate additional mediating elements, such as stress tolerance or adaptive coping, that may elucidate the mechanisms via which MF influences performance outcomes. Furthermore, these findings indicate that MF training is likely to be especially advantageous when customized to cultivate EI, as this attribute directly improves work performance. The study provides a paradigm for the integration of emotional and cognitive theories in MF research, enhancing comprehension of its diverse effects on organizational behavior.

### 5.2 Managerial Implication

This study indicates that including MF programs into organizational training can substantially enhance employees’ EI and psychological resilience. EI, as the predominant mediator, indicates that MF training should emphasize self‐regulation, empathy, and social skills, equipping employees to adeptly handle interpersonal dynamics and effectively manage workplace stressors. Programs emphasizing these skills can cultivate a supportive workplace culture that values emotional resilience and good communication, thus enhancing team cohesion and JP.

Furthermore, managers could enhance the impact of PsyCap by building a supportive work environment that reinforces optimism, resilience, and confidence. This could be achieved through mentorship programs, supportive leadership practices, and regular feedback loops that recognize and nurture employees’ strengths. By promoting both EI and PsyCap through MF training, organizations can cultivate a workforce that is both emotionally adaptive and resilient, driving sustained improvements in productivity and job satisfaction. This approach could also help organizations respond to evolving workplace challenges by creating a culture of psychological safety and adaptability.

## Author Contributions

**Hoang Cuu Long**: methodology, conceptualization, investigation, writing–review and editing, writing–original draft, project administration, supervision. **Ho Minh An**: investigation, conceptualization, software, formal analysis, visualization, validation, writing–original draft. **Pham Thi Phuong Dung**: writing–review and editing, writing–original draft, investigation, formal analysis, data curation, resources, methodology. **Nguyen Le Dinh Quy**: methodology, supervision, writing–review and editing, writing–original draft.

## Conflicts of Interest

The authors declare no conflicts of interest.

### Peer Review

The peer review history for this article is available at https://publons.com/publon/10.1002/brb3.70291.

## Data Availability

The datasets analyzed during the current study are available. The datasets generated are available from the co‐author on reasonable request. Please email leopham299@gmail.com.

## Appendix 1 Scale of Variable Measurement

### 1.1

**TABLE A1:** Cognitive and affective mindfulness scale—revised.

Attn 1. It is easy for me to concentrate on what I am doing
PreFo 2. I am preoccupied by the future (R)
Acc 3. I cannot tolerate emotional pain (R)
Acc 4. I can accept things I cannot change
Awa 5. I can usually describe how I feel at the moment in considerable detail
Attn 6. I am easily distracted (R)
PreFo 7. I am preoccupied by the past (R)
Awa 8. It's easy for me to keep track of my thoughts and feelings
Awa 9. I try to notice my thoughts without judging them
Acc 10. I am able to accept the thoughts and feelings I have
PreFo 11. I am able to focus on the present moment
Attn 12. I am able to pay close attention to one thing for a long period of time

**TABLE A2:** Emotional intelligence (WLEIS scale).

**Self‐emotion appraisal (SEA)**
SEA 1. I have a good sense of why I have certain feelings most of the time
SEA 2. I have good understanding of my own emotions
SEA 3. I really understand what I feel
SEA 4. I always know whether or not I am happy
**Others’ emotion appraisal (OEA)**
OEA 1. I always know my friends’ emotions from their behavior
OEA 2. I am a good observer of others’ emotions
OEA 3. I am sensitive to the feelings and emotions of others
OEA 4. I have good understanding of the emotions of people around me
**Use of emotion (UOE)**
UOE 1. I always set goals for myself and then try my best to achieve them
UOE 2. I always tell myself I am a competent person
UOE 3. I am a self‐motivated person
UOE 4. I would always encourage myself to try my best
**Regulation of emotion (ROE)**
ROE 1. I am able to control my temper and handle difficulties rationally
ROE 2. I am quite capable of controlling my own emotions
ROE 3. I can always calm down quickly when I am very angry
ROE 4. I have good control of my own emotions

**TABLE A3:** Psychological capital's scale.

**Hope**
HOPE 1. When I have a setback at work, I have trouble recovering from it, moving on (R)
HOPE 2. I usually manage difficulties one way or another at work
HOPE 3. I can be “on my own,” so to speak, at work if I have to
HOPE 4. I usually take stressful things at work in stride
HOPE 5. I can get through difficult times at work because I've experienced difficulty before
HOPE 6. I feel I can handle many things at a time at this job
**Self‐efficacy**
SELF 1. I feel confident analyzing a long‐term problem to find a solution
SELF 2. I feel confident in representing my work area in meetings with management
SELF 3. I feel confident contributing to discussions about the company's strategy
SELF 4. I feel confident helping to set targets/goals in my work area
SELF 5. I feel confident contacting people outside the company (e.g., suppliers, customers) to discuss problems
SELF 6. I feel confident presenting information to a group of colleagues
**Resilience**
RESI 1. If I should find myself in a jam at work, I could think of many ways to get out of it
RESI 2. At the present time, I am energetically pursuing my work goals
RESI 3. There are lots of ways around any problem
RESI 4. Right now I see myself as being pretty successful at work
RESI 5. I can think of many ways to reach my current work goals
RESI 6. At this time, I am meeting the work goals that I have set for myself
**Optimism**
OPT 1. When things are uncertain for me at work, I usually expect the best
OPT 2. If something can go wrong for me work‐wise, it will (R)
OPT 3. I always look on the bright side of things regarding my job
OPT 4. I'm optimistic about what will happen to me in the future as it pertains to work
OPT 5. In this job, things never work out the way I want them to (R)
OPT 6. I approach this job as if “every cloud has a silver lining”

**TABLE A4:** Job performance's scale.

**Contextual performance** (Organ 1988; Farh et al. 2004)
CP1. I actively help my colleagues with their work
CP2. I focus on team performance
CP3. I am courteous at work
CP4. I take measures to resolve conflict at work
CP5. I actively make suggestions to improve my company
CP6. I actively publicize my company's strengths
CP7. I manage to complete assigned work that is beyond my responsibility
CP8. I actively coordinate with my colleagues
**Task performance** (McAllister 1995; Fisher 1980)
TP1. I outperform my colleagues
TP 2. I handle emergencies well
TP 3. I achieve objectives that are assigned to me
TP 4. I am never late nor take off early from work
TP 5. I aim to attain perfection in my work
TP 6. I am prudent and seldom make mistakes

## Appendix 2 Outer Model's Result

### 2.1

**TABLE A5:** Outer model's result.

Scale		Cronbach's alpha	roh_a (CR)	roh_c (CR)	AVE
**Mindfulness**		**0.916**	**0.917**	**0.932**	**0.631**
**1. Attention (Attn)**	**0.882**	**0.722**	**0.727**	**0.887**	**0.782**
*MF1: It is easy for me to concentrate on what I am doing*	0.898				
*MF6: I am easily distracted (R)*	< 0.708				
*MF12: I am able to pay close attention to one thing for a long period of time*	0.871				
**2. Present focus (PreFo)**	**0.762**	**N/A**		**N/A**	**N/A**
*MF2: I am preoccupied by the future (R)*	< 0.708				
*MF7: I am preoccupied by the past (R)*	< 0.708				
*MF11: I am able to focus on the present moment*	1.000				
**3. Awareness (Awa)**	**0.898**	**0.784**	**0.749**	**0.888**	**0.799**
*MF5: I can usually describe how I feel at the moment in considerable detail*	< 0.708				
*MF8: It's easy for me to keep track of my thoughts and feelings*	0.898				
*MF9: I try to notice my thoughts without judging them*	0.890				
**4. Acceptance (Acc)**	**0.930**	**0.840**	**0.840**	**0.903**	**0.757**
*MF3: I cannot tolerate emotional pain (R)*	0.866				
*MF4: I can accept things I cannot change*	0.866				
*MF10: I am able to accept the thoughts and feelings I have*	0.879				
**Emotional intelligence**		**0.955**	**0.956**	**0.960**	**0.614**
**1. Self‐emotion appraisal (SEA)**	**0.936**	**0.889**	**0.893**	**0.924**	**0.752**
*SEA 1. I have a good sense of why I have certain feelings most of the time*	0.897				
*SEA 2. I have good understanding of my own emotions*	0.886				
*SEA 3. I really understand what I feel*	0.889				
*SEA 4. I always know whether or not I am happy*	0.792				
**2. Others’ emotion appraisal (OEA)**	**0.909**	**0.819**	**0.822**	**0.892**	**0.734**
*OEA 1. I always know my friends’ emotions from their behavior*	< 0.708				
*OEA 2. I am a good observer of others’ emotions*	0.841				
*OEA 3. I am sensitive to the feelings and emotions of others*	0.872				
*OEA 4. I have good understanding of the emotions of people around me*	0.857				
**3. Use of emotion (UOE)**	**0.884**	**0.905**	**0.906**	**0.934**	**0.779**
*UOE 1. I always set goals for myself and then try my best to achieve them*	0.896				
*UOE 2. I always tell myself I am a competent person*	0.896				
*UOE 3. I am a self‐motivated person*	0.904				
*UOE 4. I would always encourage myself to try my best*	0.832				
4. **Regulation of emotion (ROE)**	**0.918**	**0.851**	**0.852**	**0.899**	**0.691**
*ROE 1. I am able to control my temper and handle difficulties rationally*	0.859				
*ROE 2. I am quite capable of controlling my own emotions*	0.847				
*ROE 3. I can always calm down quickly when I am very angry*	0.808				
*ROE 4. I have good control of my own emotions*	0.809				
**Psychological capital (PsyCap)**		**0.969**	**0.970**	**0.972**	**0.620**
**1. Hope (HOPE)**	**0.955**	**0.882**	**0.882**	**0.914**	**0.680**
*HOPE 1. When I have a setback at work, I have trouble recovering from it, moving on (R)*	< 0.708				
*HOPE 2. I usually manage difficulties one way or another at work*	0.853				
*HOPE 3. I can be “on my own,” so to speak, at work if I have to*	0.822				
*HOPE 4. I usually take stressful things at work in stride*	0.833				
*HOPE 5. I can get through difficult times at work because I've experienced difficulty before*	0.816				
*HOPE 6. I feel I can handle many things at a time at this job*	0.797				
**2. Self‐efficacy (SELF)**	**0.943**	**0.900**	**0.902**	**0.923**	**0.668**
*SELF 1. I feel confident analyzing a long‐term problem to find a solution*	0.819				
*SELF 2. I feel confident in representing my work area in meetings with management*	0.850				
*SELF 3. I feel confident contributing to discussions about the company's strategy*	0.778				
*SELF 4. I feel confident helping to set targets/goals in my work area*	0.837				
*SELF 5. I feel confident contacting people outside the company (e.g., suppliers, customers) to discuss problems*	0.807				
*SELF 6. I feel confident presenting information to a group of colleagues*	0.810				
**3. Resilience (RESI)**	**0.958**	**0.899**	**0.901**	**0.922**	**0.664**
*RESI 1. If I should find myself in a jam at work, I could think of many ways to get out of it*	0.806				
*RESI 2. At the present time, I am energetically pursuing my work goals*	0.759				
*RESI 3. There are lots of ways around any problem*	0.859				
*RESI 4. Right now I see myself as being pretty successful at work*	0.825				
*RESI 5. I can think of many ways to reach my current work goals*	0.800				
*RESI 6. At this time, I am meeting the work goals that I have set for myself*	0.838				
**4. Optimism (OPT)**	**0.931**	**0.906**	**0.907**	**0.934**	**0.780**
*OPT 1. When things are uncertain for me at work, I usually expect the best*	0.874				
*OPT 2. If something can go wrong for me work‐wise, it will (R)*	< 0.708				
*OPT 3. I always look on the bright side of things regarding my job*	0.889				
*OPT 4. I'm optimistic about what will happen to me in the future as it pertains to work*	0.858				
*OPT 5. In this job, things never work out the way I want them to (R)*	< 0.708				
*OPT 6. I approach this job as if “every cloud has a silver lining”*	0.912				
**Job performance (JP)**		**0.953**	**0.954**	**0.958**	**0.621**
**1. Contextual performance (CP)**	**0.982**	**0.924**	**0.926**	**0.938**	**0.654**
*CP1. I actively help my colleagues with their work*	0.884				
*CP2. I focus on team performance*	0.777				
*CP3. I am courteous at work*	0.780				
*CP4. I take measures to resolve conflict at work*	0.832				
*CP5. I actively make suggestions to improve my company*	0.788				
*CP6. I actively publicize my company's strengths*	0.741				
*CP7. I manage to complete assigned work that is beyond my responsibility*	0.817				
*CP8. I actively coordinate with my colleagues*	0.841				
**2. Task performance (TP)**	**0.967**	**0.892**	**0.894**	**0.917**	**0.649**
*TP1. I outperform my colleagues*	0.787				
*TP 2. I handle emergencies well*	0.817				
*TP 3. I achieve objectives that are assigned to me*	0.849				
*TP 4. I am never late nor take off early from work*	0.759				
*TP 5. I aim to attain perfection in my work*	0.838				
*TP 6. I am prudent and seldom make mistakes*	0.783				

## References

[brb370291-bib-0001] Organ, D. W. 1988. Organizational Citizenship Behavior—The Good Soldier Syndrome. Lexington, MA: Lexington Books.

[brb370291-bib-0002] Aboagye, A. K. , B. Dai , and E. K. Bakpa . 2022. “Influence of Risk Perception on Task and Contextual Performance: A Case of Work‐Related Musculoskeletal Disorders in Nurses.” Evaluation & the Health Professions 45, no. 2: 126–136. 10.1177/0163278720975071. 33291982

[brb370291-bib-0004] Ali, M. , A. N. Khan , M. M. Khan , A. S. Butt , and S. H. H. Shah . 2022. “Mindfulness and Study Engagement: Mediating Role of Psychological Capital and Intrinsic Motivation.” Journal of Professional Capital and Community 7, no. 2: 144–158. 10.1108/JPCC-02-2021-0013.

[brb370291-bib-0005] Alonazi, W. B. 2020. “The Impact of Emotional Intelligence on Job Performance During covid‐19 Crisis: A Cross‐Sectional Analysis.” Psychology Research and Behavior Management 13: 749–757. 10.2147/PRBM.S263656. 33061691 PMC7520462

[brb370291-bib-0006] Alsufyani, A. M. , A. E. Aboshaiqah , F. A. Alshehri , and Y. M. Alsufyani . 2022. “Impact of Emotional Intelligence on Work Performance: The Mediating Role of Occupational Stress Among Nurses.” Journal of Nursing Scholarship 54, no. 6: 738–749. 10.1111/jnu.12790. 35650636

[brb370291-bib-0011] Berdida, D. J. E. , V. Lopez , and R. A. N. Grande . 2023. “Nursing Students' Perceived Stress, Social Support, Self‐Efficacy, Resilience, Mindfulness and Psychological Well‐Being: A Structural Equation Model.” International Journal of Mental Health Nursing 32, no. 5: 1390–1404. 10.1111/inm.13179. 37249199

[brb370291-bib-0013] Bhatti, M. A. , N. Mat , and A. S. Juhari . 2018. “Effects of Job Resources Factors on Nurses Job Performance (Mediating Role of Work Engagement).” International Journal of Health Care Quality Assurance 31, no. 8: 1000–1013. 10.1108/IJHCQA-07-2017-0129. 30415625

[brb370291-bib-0014] Bi, Y. , and X. Ye . 2021. “The Effect of Trait Mindfulness on Teachers' Emotional Exhaustion: The Chain Mediating Role of Psychological Capital and Job Engagement.” Healthcare (Basel) 9, no. 11: 1527. 10.3390/healthcare9111527. 34828573 PMC8619463

[brb370291-bib-0016] Bockorny, K. , and C. M. Youssef‐Morgan . 2019. “Entrepreneurs' Courage, Psychological Capital, and Life Satisfaction.” Frontiers in Psychology 10: 789–789. 10.3389/fpsyg.2019.00789. 31024410 PMC6461011

[brb370291-bib-0017] Borman, W. C. , and S. J. Motowidlo . 1993. “Expanding the Criterion Domain to Include Elements of Contextual Performance.” In Personnel Selection in Organizations, edited by N. Schmitt and W. C. Borman , 71–98. San Francisco, CA: Jossey‐Bass.

[brb370291-bib-0019] Bramantya, A. , and M. Muafi . 2022. “The Effect of Perceived Organizational Support and Psychological Capital on Work Performance Mediated by Organizational Citizenship Behavior.” International Journal of Research in Business and Social Science 11, no. 6: 229–240. 10.20525/ijrbs.v11i6.2040.

[brb370291-bib-0021] Chauhan, R. , V. Kaul , and N. Maheshwari . 2022. “Impact of Emotional Intelligence on Job Performance of Nurses With Mediating Effect of Job Satisfaction.” Asia Pacific Journal of Health Management 17, no. 2: 1–8. 10.24083/apjhm.v17i2.1257.

[brb370291-bib-0025] Cohen, J. 1988. Statistical Power Analysis for the Behavioral Sciences. New York: Routledge. 10.4324/9780203771587.

[brb370291-bib-0026] Coleman, C. N. , and K. F. Coleman . 2019. Mindfulness for the High Performance World*: A* Practical, Skill‐Based Approach to Developing and Sustaining Mindfulness*, Equanimity and Balance* . Cham: Springer International Publishing. 10.1007/978-3-030-18582-4.

[brb370291-bib-0027] Dai, Z. , H. Wang , W. Xiao , et al. 2022. “The Association of Mindfulness and Psychological Well‐Being Among Individuals Who Have Recovered From COVID‐19 in Jianghan District, Wuhan, China: A Cross‐Sectional Study.” Journal of Affective Disorders 319: 437–445. 10.1016/j.jad.2022.09.062. 36162667 PMC9502442

[brb370291-bib-0028] Dane, E. 2011. “Paying Attention to Mindfulness and Its Effects on Task Performance in the Workplace.” Journal of Management 37, no. 4: 997–1018. 10.1177/0149206310367948.

[brb370291-bib-0031] Deshpande, P. , and A. P. Srivastava . 2023. “A Study to Explore the Linkage Between Green Training and Sustainable Organizational Performance Through Emotional Intelligence and Green Work Life Balance.” European Journal of Training and Development 47, no. 5/6: 615–634. 10.1108/EJTD-11-2021-0182.

[brb370291-bib-0033] Dirzyte, A. , A. Perminas , and E. Biliuniene . 2021. “Psychometric Properties of Satisfaction With Life Scale (SWLS) and Psychological Capital Questionnaire (PCQ‐24) in the Lithuanian Population.” International Journal of Environmental Research and Public Health 18, no. 5: 2608. 10.3390/ijerph18052608. 33807777 PMC7967519

[brb370291-bib-0034] Drolet, A. L. , and D. G. Morrison . 2001. “Do We Really Need Multiple‐Item Measures in Service Research?” Journal of Service Research: JSR 3, no. 3: 196–204. 10.1177/109467050133001.

[brb370291-bib-0035] Enkema, M. C. , L. McClain , E. R. Bird , M. A. Halvorson , and M. E. Larimer . 2020. “Associations Between Mindfulness and Mental Health Outcomes: A Systematic Review of Ecological Momentary Assessment Research.” Mindfulness 11, no. 11: 2455–2469. 10.1007/s12671-020-01442-2. 35694042 PMC9187214

[brb370291-bib-0037] Fei, J. , Y. Hu , L. Liang , C. Meng , and S. Mei . 2023. “Exploring the Impact of Emotional and Cognitive Factors on Anxiety Symptoms of Chinese Adolescents: A Serial Mediation Study.” International Journal of Mental Health and Addiction 22: 1–15. 10.1007/s11469-022-01004-8. PMC9838377 36688112

[brb370291-bib-0038] Feldman, G. , A. Hayes , S. Kumar , J. Greeson , and J.‐P. Laurenceau . 2007. “Mindfulness and Emotion Regulation: The Development and Initial Validation of the Cognitive and Affective Mindfulness Scale‐Revised (CAMS‐R).” Journal of Psychopathology and Behavioral Assessment 29, no. 3: 177–190. 10.1007/s10862-006-9035-8.

[brb370291-bib-0040] Fiaz, S. , and S. Muhammad Fahim . 2023. “The Influence of High‐Quality Workplace Relational Systems and Mindfulness on Employee Work Engagement at the Time of Crises.” Heliyon 9, no. 4: e15523. 10.1016/j.heliyon.2023.e15523. 37128340 PMC10148041

[brb370291-bib-0041] Finch, J. , L. J. Farrell , and A. M. Waters . 2020. “Searching for the HERO in Youth: Does Psychological Capital (PsyCap) Predict Mental Health Symptoms and Subjective Wellbeing in Australian School‐Aged Children and Adolescents?” Child Psychiatry and Human Development 51, no. 6: 1025–1036. 10.1007/s10578-020-01023-3. 32666426 PMC7358995

[brb370291-bib-0042] Fisher, C. D. 1980. “On the Dubious Wisdom of Expecting Job Satisfaction to Correlate With Performance.” Academy of Management Review 5, no. 4: 607–612. 10.2307/257468.

[brb370291-bib-0046] Garland, E. L. , N. A. Farb , P. R. Goldin , and B. L. Fredrickson . 2015. “Mindfulness Broadens Awareness and Builds Eudaimonic Meaning: A Process Model of Mindful Positive Emotion Regulation.” Psychological Inquiry 26, no. 4: 293–314. 10.1080/1047840X.2015.1064294. 27087765 PMC4826727

[brb370291-bib-0047] George, O. J. , S. E. Okon , and G. O. Akaighe . 2023. “Psychological Capital and Work Engagement Among Employees in the Nigerian Public Sector: The Mediating Role of Emotional Intelligence.” International Journal of Public Administration 46, no. 6: 445–453. 10.1080/01900692.2021.2001010.

[brb370291-bib-0050] Gong, Z. , Y. Chen , and Y. Wang . 2019. “The Influence of Emotional Intelligence on Job Burnout and Job Performance: Mediating Effect of Psychological Capital.” Frontiers in Psychology 10: 2707. 10.3389/fpsyg.2019.02707. 31920783 PMC6916327

[brb370291-bib-0051] Good, D. J. , C. J. Lyddy , T. M. Glomb , et al. 2016. “Contemplating Mindfulness at Work: An Integrative Review.” Journal of Management 42, no. 1: 114–142. 10.1177/0149206315617003.

[brb370291-bib-0052] Gordani, Y. , and M. Sadeghzadeh . 2023. “Mindfulness and the Mediating Role of Psychological Capital in Predicting the Foreign Language Anxiety.” Journal of Psycholinguistic Research 52, no. 5: 1785–1797. 10.1007/s10936-023-09938-3. 37204673

[brb370291-bib-0055] Hair, J. F. , G. T. M. Hult , C. M. Ringle , and M. Sarstedt . 2022. A Primer on Partial Least Squares Structural Equation Modeling (PLS‐SEM). Los Angeles, CA: SAGE Publications, Inc.

[brb370291-bib-0056] Hair, J. F. , J. J. Risher , M. Sarstedt , and C. M. Ringle . 2019. “When to Use and How to Report the Results of PLS‐SEM.” European Business Review 31, no. 1: 2–24. 10.1108/EBR-11-2018-0203.

[brb370291-bib-0057] Hair, J. F. , and M. Sarstedt . 2021. “Explanation plus Prediction—The Logical Focus of Project Management Research.” Project Management Journal 52, no. 4: 319–322. 10.1177/8756972821999945.

[brb370291-bib-0059] Hameed, A. , M. G. Khwaja , and U. Zaman . 2023. “Configuring Optimal Contextual Performance and Task Performance in Offshore Business Processing Organizations.” Business Process Management Journal 29, no. 1: 285–307. 10.1108/BPMJ-07-2022-0330.

[brb370291-bib-0061] Hayduk, L. A. , and L. Littvay . 2012. “Should Researchers Use Single Indicators, Best Indicators, or Multiple Indicators in Structural Equation Models?” BMC Medical Research Methodology 12, no. 1: 159. 10.1186/1471-2288-12-159. 23088287 PMC3506474

[brb370291-bib-0064] Himes, L. , N. A. Hubbard , G. B. Maruthy , J. Gallagher , M. P. Turner , and B. Rypma . 2021. “The Relationship Between Trait Mindfulness and Emotional Reactivity Following Mood Manipulation.” Mindfulness 12, no. 1: 170–185. 10.1007/s12671-020-01510-7.

[brb370291-bib-0067] Huang, H. , L. Gao , X. Deng , and H. Fu . 2022. “The Relationship Between Emotional Intelligence and Expatriate Performance in International Construction Projects.” Psychology Research and Behavior Management 15: 3825–3843. 10.2147/PRBM.S387287. 36578282 PMC9791953

[brb370291-bib-0068] Huang, S. (Sam) , Z. Yu , Y. Shao , M. Yu , and Z. Li . 2021. “Relative Effects of Human Capital, Social Capital and Psychological Capital on Hotel Employees' Job Performance.” International Journal of Contemporary Hospitality Management 33, no. 2: 490–512. 10.1108/IJCHM-07-2020-0650.

[brb370291-bib-0070] Iliceto, P. , and E. Fino . 2017. “The Italian Version of the Wong‐Law Emotional Intelligence Scale (WLEIS‐I): A Second‐Order Factor Analysis.” Personality and Individual Differences 116: 274–280. 10.1016/j.paid.2017.05.006.

[brb370291-bib-0071] Jacobs, I. , and A. Wollny . 2022. “Personal Values, Trait Emotional Intelligence, and Mental Health Problems.” Scandinavian Journal of Psychology 63, no. 2: 155–163. 10.1111/sjop.12785. 34734412

[brb370291-bib-0072] Janssen, E. , I. Van Strydonck , A. Decuypere , A. Decramer , and M. Audenaert . 2020. “How to Foster Nurses' Well‐Being and Performance in the Face of Work Pressure? The Role of Mindfulness as Personal Resource.” Journal of Advanced Nursing 76, no. 12: 3495–3505. 10.1111/jan.14563. 32989794

[brb370291-bib-0073] Jeong, J. , and J. H. Lee . 2022. “Customer Mistreatment, Employee Depression, and Organizational Citizenship Behavior: Emotional Intelligence as a Moderator.” Social Behavior and Personality 50, no. 3: 90–98. 10.2224/sbp.11167.

[brb370291-bib-0074] Jiao, Y. , X. Zhang , S. Lu , Z. Wu , and Y. Deng . 2022. “Research on the Influence of Team Psychological Capital on Team Members' Work Performance.” Frontiers in Psychology 13: 1072158. 10.3389/fpsyg.2022.1072158. 36582334 PMC9792663

[brb370291-bib-0082] Kotzé, M. 2018. “The Influence of Psychological Capital, Self‐Leadership, and Mindfulness on Work Engagement.” South African Journal of Psychology 48, no. 2: 279–292. 10.1177/0081246317705812.

[brb370291-bib-0083] Kou, H. , T. Bi , S. Chen , et al. 2022. “The Impact of Mindfulness Training on Supportive Communication, Emotional Intelligence, and Human Caring Among Nursing Students.” Perspectives in Psychiatric Care 58, no. 4: 2552–2561. 10.1111/ppc.13093. 35426149

[brb370291-bib-0086] Levin, K. A. 2006. “Study Design III: Cross‐Sectional Studies.” Evidence‐Based Dentistry 7, no. 1: 24–25. 10.1038/sj.ebd.6400375. 16557257

[brb370291-bib-0087] Li, Y. 2018. “Linking Protean Career Orientation to Well‐Being: The Role of Psychological Capital.” Career Development International 23, no. 2: 178–196. 10.1108/CDI-07-2017-0132.

[brb370291-bib-0088] Liao, S.‐H. , D.‐C. Hu , and Y.‐C. Huang . 2022. “Employee Emotional Intelligence, Organizational Citizen Behavior and Job Performance: A Moderated Mediation Model Investigation.” Employee Relations 44, no. 5: 1109–1126. 10.1108/ER-11-2020-0506.

[brb370291-bib-0089] Lima, S. , C. Garrett , J. C. Machado , M. Vilaça , and M. G. Pereira . 2020. “Quality of Life in Patients With Mild Alzheimer Disease: The Mediator Role of Mindfulness and Spirituality.” Aging & Mental Health 24, no. 12: 2103–2110. 10.1080/13607863.2019.1650891. 31411042

[brb370291-bib-0090] Lin, C.‐Y. , C.‐K. Huang , H.‐X. Li , T.‐W. Chang , and Y.‐C. Hsu . 2022. “Will They Stay or Leave? Interplay of Organizational Learning Culture and Workplace Mindfulness on Job Satisfaction and Turnover Intentions.” Public Personnel Management 51, no. 1: 24–47. 10.1177/0091026021991581.

[brb370291-bib-0095] Luthans, F. , C. M. Youssef , and B. J. Avolio . 2007. Psychological Capital Developing the Human Competitive Edge. Oxford: Oxford University Press.

[brb370291-bib-0096] Lye, A. J. , P. Y. Liew , H. Mohd Fadzil , and C. C. Foong . 2023. “Effect of Emotional Intelligence and Demographic Characteristics on Psychological Capital Among Chemical Engineering Students.” Journal of Chemical Education 100, no. 2: 479–488. 10.1021/acs.jchemed.2c00105.

[brb370291-bib-0170] Mayer, J. D. , and P. Salovey . 1997. “What is emotional intelligence? Emotional development and emotional intelligence: Implications for educators.” New York: Basic Book PP, 3–31.

[brb370291-bib-0099] McAllister, D. J. 1995. “Affect‐ and Cognition‐Based Trust as Foundations for Interpersonal Cooperation in Organizations.” Academy of Management Journal 38, no. 1: 24–59. 10.5465/256727.

[brb370291-bib-0101] Mesquita, B. L. B. , F. Ribeirinho Soares , and M. Fraga , et al. 2023. “Mindfulness‐Based Interventions for Anxiety and Depression.” European Psychiatry 66, no. S1: S948. 10.1192/j.eurpsy.2023.2009.

[brb370291-bib-0102] Mittal, S. 2021. “Ability‐Based Emotional Intelligence and Career Adaptability: Role in Job‐Search Success of University Students.” Higher Education, Skills and Work‐Based Learning 11, no. 2: 454–470. 10.1108/HESWBL-10-2019-0145.

[brb370291-bib-0103] Mjoli, T. Q. , and J. K. Aderibigbe . 2019. “Relationship Between Occupational Stress, Organisational Citizenship Behaviour, Psychological Capital and Emotional Intelligence Among Nigerian Employees.” African Journal of Business and Economic Research 14, no. 1: 85–111. 10.31920/1750-4562/2019/v14n1a5.

[brb370291-bib-0104] Moeller, R. W. , M. Seehuus , and V. Peisch . 2020. “Emotional Intelligence, Belongingness, and Mental Health in College Students.” Frontiers in Psychology 11: 93–93. 10.3389/fpsyg.2020.00093. 32076414 PMC7006433

[brb370291-bib-0105] Ng, H. H. , C. W. Wu , F. Huang , et al. 2023. “Enhanced Electroencephalography Effective Connectivity in Frontal Low‐Gamma Band Correlates of Emotional Regulation After Mindfulness Training.” Journal of Neuroscience Research 101, no. 6: 901–915. 10.1002/jnr.25168. 36717762

[brb370291-bib-0108] Oh, H. , and J. Jang . 2020. “The Role of Team‐Member Exchange: Restaurant Servers' Emotional Intelligence, Job Performance, and Tip Size.” Journal of Human Resources in Hospitality & Tourism 19, no. 1: 43–61. 10.1080/15332845.2020.1672248.

[brb370291-bib-0110] Paliga, M. , B. Kozusznik , A. Pollak , and E. Sanecka . 2022. “The Relationships of Psychological Capital and Influence Regulation With Job Satisfaction and Job Performance.” PLoS ONE 17, no. 8: e0272412. 10.1371/journal.pone.0272412. 35943992 PMC9362931

[brb370291-bib-0111] Pan, B. , H. Wu , and X. Zhang . 2022. “The Effect of Trait Mindfulness on Subjective Well‐Being of Kindergarten Teachers: The Sequential Mediating Roles of Emotional Intelligence and Work–Family Balance.” Psychology Research and Behavior Management 15: 2815–2830. 10.2147/PRBM.S381976. 36199972 PMC9529014

[brb370291-bib-0114] Preacher, K. J. , and A. F. Hayes . 2004. “SPSS and SAS Procedures for Estimating Indirect Effects in Simple Mediation Models.” Behavior Research Methods 36, no. 4: 717–731. 10.3758/bf03206553 15641418

[brb370291-bib-0115] Preacher, K. J. , and A. F. Hayes . 2008. “Asymptotic and Resampling Strategies for Assessing and Comparing Indirect Effects in Multiple Mediator Models.” Behavior Research Methods 40, no. 3: 879–891. 18697684 10.3758/brm.40.3.879

[brb370291-bib-0116] Pressman, A. , M. Dolginsky , B. Stahl , et al. 2017. “Fidelity Measurement of a Mindfulness‐Based Stress Reduction Intervention in a Randomized Controlled Feasibility Trial for Migraine Patients.” Journal of Patient‐Centered Research and Reviews 4, no. 3: 199–200. 10.17294/2330-0698.1571.

[brb370291-bib-0122] Sarwar, U. , M. Aamir , Y. Bichao , and Z. Chen . 2023. “Authentic Leadership, Perceived Organizational Support, and Psychological Capital: Implications for Job Performance in the Education Sector.” Frontiers in Psychology 13: 1084963. 10.3389/fpsyg.2022.1084963. 36698565 PMC9869258

[brb370291-bib-0125] Schutte, N. S. , S.‐L. Keng , and M. W. L. Cheung . 2021. “Emotional Intelligence Mediates the Connection Between Mindfulness and Gratitude: A Meta‐Analytic Structural Equation Modeling Study.” Mindfulness 12, no. 11: 2613–2623. 10.1007/s12671-021-01725-2.

[brb370291-bib-0128] Shahbaz, W. , and J. Parker . 2022. “Workplace Mindfulness: An Integrative Review of Antecedents, Mediators, and Moderators.” Human Resource Management Review 32, no. 3: 100849. 10.1016/j.hrmr.2021.100849.

[brb370291-bib-0130] Shakeel, U. , and F. S. Khan . 2023. “Influence of Emotional Intelligence on Employees' Performance at Work: An Evidence From Indian Banks.” Journal of Statistics & Management Systems 26, no. 3: 561–571. 10.47974/JSMS-1047.

[brb370291-bib-0131] Shan, H. , Z. Ishak , and L. Fan . 2022. “The Higher the Life Satisfaction, the Better the Psychological Capital? Life Satisfaction and Psychological Capital: A Moderated Mediation Model.” Frontiers in Psychology 12: 772129. 10.3389/fpsyg.2021.772129. 35498151 PMC9045006

[brb370291-bib-0132] Sharma, S. , and V. Tiwari . 2023. “Emotional Intelligence and Career Success: Does Resilience Matter?” Global Business and Organizational Excellence 42, no. 6: 138–153. 10.1002/joe.22196.

[brb370291-bib-0134] Siegel, R. D. , C. K. Germer , and A. Olendzki . n.d. “Mindfulness: What Is It? Where Did It Come from?” In Clinical Handbook of Mindfulness, 17–35. New York: Springer. 10.1007/978-0-387-09593-6_2.

[brb370291-bib-0140] Song, Z. , B. Pan , and Y. Wang . 2021. “Can Trait Mindfulness Improve Job Satisfaction? The Relationship Between Trait Mindfulness and Job Satisfaction of Preschool Teachers: The Sequential Mediating Effect of Basic Psychological Needs and Positive Emotions.” Frontiers in Psychology 12: 788035–788035. 10.3389/fpsyg.2021.788035. 34966337 PMC8710905

[brb370291-bib-0141] Sood, S. , and D. Puri . 2022. “Psychological Capital and Positive Mental Health of Student‐Athletes: Psychometric Properties of the Sport Psychological Capital Questionnaire.” Current Psychology (New Brunswick, N.J.) 42, no. 25: 21759–21774. 10.1007/s12144-022-03272-y.

[brb370291-bib-0142] Syeda, M. M. , and J. J. W. Andrews . 2021. “Mindfulness‐Based Cognitive Therapy as a Targeted Group Intervention: Examining Children's Changes in Anxiety Symptoms and Mindfulness.” Journal of Child and Family Studies 30, no. 4: 1002–1015. 10.1007/s10826-021-01924-4.

[brb370291-bib-0144] Tang, Y. , Y.‐F. Shao , and Y.‐J. Chen . 2019. “Assessing the Mediation Mechanism of Job Satisfaction and Organizational Commitment on Innovative Behavior: The Perspective of Psychological Capital.” Frontiers in Psychology 10: 2699. 10.3389/fpsyg.2019.02699. 31920781 PMC6928100

[brb370291-bib-0146] Thuy, N. T. T. , and B.‐A. Phinaitrup . 2023. “The Effect of Public Service Motivation on Job Performance of Public Servants in Vietnam: The Role of Mediation of Job Satisfaction and Person‐Organization Fit.” International Journal of Public Administration 46, no. 5: 326–343. 10.1080/01900692.2021.1995747.

[brb370291-bib-0148] Tiwari, S. , and P. Garg . 2019. “Promoting Basic Need Satisfaction at Workplace: The Relevance of Mindfulness in Support of Job Performance of Employees.” Jindal Journal of Business Research 8, no. 1: 1–15. 10.1177/2278682118785812.

[brb370291-bib-0149] Tüzün, I. K. , F. Çetin , and H. N. Basim . 2018. “Improving Job Performance Through Identification and Psychological Capital.” International Journal of Productivity and Performance Management 67, no. 1: 155–170. 10.1108/IJPPM-03-2016-0060.

[brb370291-bib-0152] Wang, H. , and L. Lei . 2023. “Proactive Personality and Job Satisfaction: Social Support and Hope as Mediators.” Current Psychology (New Brunswick, N.J.) 42, no. 1: 126–135. 10.1007/s12144-021-01379-2.

[brb370291-bib-0154] Wang, Z. , C. C. H. Yip , D. C. K. Leung , and K. K. S. Chan . 2023. “The Impact of Mindfulness on Stigma Stress and Well‐Being Among Individuals With Mental Disorders.” Mindfulness 14, no. 4: 808–817. 10.1007/s12671-023-02111-w.

[brb370291-bib-0157] Whitaker, R. C. , A. N. Herman , T. Dearth‐Wesley , et al. 2019. “The Association of Fatigue With Dispositional Mindfulness: Relationships by Levels of Depressive Symptoms, Sleep Quality, Childhood Adversity, and Chronic Medical Conditions.” Preventive Medicine 129: 105873. 10.1016/j.ypmed.2019.105873. 31644898

[brb370291-bib-0161] Wong, C.‐S. , and K. S. Law . 2002. “The Effects of Leader and Follower Emotional Intelligence on Performance and Attitude: An Exploratory Study.” Leadership Quarterly 13, no. 3: 243–274. 10.1016/S1048-9843(02)00099-1.

[brb370291-bib-0162] Xiang, Y. , X. Dong , J. Zhao , Q. Li , J. Zhao , and W. Zhang . 2021. “The Relationship Between Mindfulness and Envy: The Mediating Role of Emotional Intelligence.” PsyCh Journal 10, no. 6: 898–904. 10.1002/pchj.493. 34755495

[brb370291-bib-0163] Xie, C. , X. Li , Y. Zeng , and X. Hu . 2021. “Mindfulness, Emotional Intelligence and Occupational Burnout in Intensive Care Nurses: A Mediating Effect Model.” Journal of Nursing Management 29, no. 3: 535–542. 10.1111/jonm.13193. 33103273

[brb370291-bib-0164] Xie, J. , K. Ifie , and T. Gruber . 2022. “The Dual Threat of COVID‐19 to Health and Job Security—Exploring the Role of Mindfulness in Sustaining Frontline Employee‐Related Outcomes.” Journal of Business Research 146: 216–227. 10.1016/j.jbusres.2022.03.030. 35340762 PMC8934737

[brb370291-bib-0169] Zhu, X. , W. Kunaviktikul , S. Sirakamon , K. Abhicharttibutra , and S. Turale . 2021. “A Causal Model of Thriving at Work in Chinese Nurses.” International Nursing Review 68, no. 4: 444–452. 10.1111/inr.12671. 33682932

